# Stone Heart Syndrome After Aortic Valve Replacement for Severe Aortic Stenosis: A Case Report

**DOI:** 10.7759/cureus.101968

**Published:** 2026-01-21

**Authors:** Lampriani Papageorgiou

**Affiliations:** 1 Department of Cardiovascular and Thoracic Surgery Anaesthesiology, Nicosia General Hospital, Nicosia, CYP

**Keywords:** aortic valve replacement, cardioplegia, cardiopulmonary bypass, extracorporeal membrane oxygenation, ischemic myocardial contracture, myocardial ischemia, stone heart syndrome

## Abstract

Stone heart syndrome (SHS) is a rare but catastrophic complication of cardiac surgery, characterized by irreversible ischemic myocardial contracture following prolonged global myocardial ischemia, and is almost uniformly fatal once established. We report the case of a 55-year-old woman with severe symptomatic aortic stenosis who underwent elective mechanical aortic valve replacement. Preoperative echocardiography showed preserved ventricular dimensions with low-normal systolic function. Due to technical difficulties during mini-sternotomy, a prolonged aortic cross-clamp time was required with repeated cardioplegia. Failure to wean from cardiopulmonary bypass was followed by complete left ventricular akinesia, immobile left-sided valves, and minimal right ventricular activity despite maximal support. Full sternotomy revealed a rigid, non-compliant myocardium consistent with SHS. Central veno-arterial extracorporeal membrane oxygenation was instituted without myocardial recovery. The patient died from multiorgan failure on postoperative day three. SHS results from profound myocardial ischemia, causing adenosine triphosphate depletion, calcium overload, and irreversible actin-myosin binding. Prevention, achieved through meticulous myocardial protection and strict minimization of ischemic time, remains the only effective strategy against this devastating and irreversible complication of cardiac surgery.

## Introduction

In 1972, Cooley and colleagues first described a rare and catastrophic condition occurring after cardiopulmonary bypass, characterized by a rigid, non-contractile heart arrested in systole. This fatal clinical complication was characterized as “stone heart” syndrome (SHS) [1]. Unfortunately, this condition does not respond to vigorous manual massage, inotropic agents, adrenergic blockers, mechanical devices, or any temporary measures, and its fatal course cannot be altered or reversed. On palpation, the heart resembles a tetanic contraction of striated muscle. The myocardium undergoes irreversible actin-myosin cross-bridge formation due to adenosine triphosphate (ATP) depletion, causing the heart to become rigid, contracted, and non-functional [2].

Its incidence was first described to be 25 cases every 10,000 heart surgeries [1]. In the reported incidence, severe left ventricular hypertrophy was usually present as a result of severe aortic stenosis [1]. Other predisposing factors include chronic congestive heart failure and myocardial hypertrophy with fibrosis secondary to long-standing aortic valve disease [3], pulmonary hypertension, and conduction abnormalities [4]. Aortic cross-clamp time is an independent factor that increases the risk of SHS accordingly because of the prolongation of cardiac ischemia time [5].

SHS is mostly the result of prolonged cardiac arrest, causing severe myocardial hypoxia and anaerobic metabolism, which lead to ischemic contracture [6]. Despite extensive research, the exact causative mechanisms that lead to this irreversible cell damage remain unclear [7]. Energy depletion, specifically depletion of myocardial ATP stores, and an imbalance between myocardial energy demand and supply represent an oversimplified explanation for this complex phenomenon [3,4,8]. Nevertheless, beta adrenergic blockage reduces the risk of SHS [8-11], so as hypothermia especially if applied before aortic cross-clamping [3,7-9,11]. Cool cardioplegia can prevent but not treat this condition [4].

According to studies, in SHS, there is severe band necrosis of the subendocardial half of the entire circumference of both the left and the right ventricles, also referred to as myofibrillar degeneration or hemorrhagic infarction of myocardial muscle cells, while the coronary arteries remain patent. The outer subepicardial half of the myocardium is usually spared [8]. It is speculated that contraction band necrosis could be the result of reflow, specifically of reperfusion post a period of non-perfusion to the heart [8], though this theory has been contradicted by recent literature. There have been reports of the syndrome in newborns who have just entered cardiopulmonary bypass, much before any reperfusion phenomena [12].

Although the incidence of SHS has significantly declined due to advances in myocardial protection, cardioplegia techniques, and perioperative monitoring, isolated cases continue to be reported in modern practice [5,7,12,13]. This rare yet fatal condition of cardiothoracic surgery is most commonly associated with prolonged aortic cross-clamp time, severe myocardial hypertrophy, and inadequate myocardial protection. Once established, the condition is refractory to pharmacological therapy, mechanical circulatory support, and resuscitative measures [3,4]. We present a fatal case of SHS following aortic valve replacement in a patient with severe aortic stenosis, highlighting the importance of meticulous surgical planning and ischemic time minimization.

## Case presentation

A 55-year-old woman was admitted for elective aortic valve replacement due to symptomatic severe aortic stenosis. Her medical history was significant for morbid obesity (body mass index: 46.9 kg/m²), hypertension, type 2 diabetes mellitus, and dyslipidemia. Preoperative laboratory investigations were within normal limits. Electrocardiography showed a normal sinus rhythm, and chest radiography was unremarkable.

Preoperative transoesophageal echocardiography demonstrated severe aortic stenosis with a peak gradient of 104 mmHg and an aortic valve area of 0.4 cm² in a quadricuspid native valve. Left ventricular dimensions were normal with low-normal systolic function (ejection fraction: 50-55%) and concentric myocardial thickening. Mild mitral valve stenosis and regurgitation were also noted. Coronary angiography revealed non-obstructive coronary arteries (Videos 1-4).

**Video 1:** Coronary angiogram of a case with severe aortic stenosis requiring aortic valve replacement (part 1). Non-obstructive coronary artery disease was present.

**Video 2:** Coronary angiogram of a case with severe aortic stenosis requiring aortic valve replacement (part 2). Non-obstructive coronary artery disease was present.

**Video 3:** Coronary angiogram of a case with severe aortic stenosis requiring aortic valve replacement (part 3). Non-obstructive coronary artery disease was present.

**Video 4:** Coronary angiogram of a case with severe aortic stenosis requiring aortic valve replacement (part 4). Non-obstructive coronary artery disease was present.

The patient underwent general anesthesia and a mini-sternotomy. Cardiopulmonary bypass was established with aortic and right atrial cannulation, and antegrade cold cardioplegia was administered. A mechanical bileaflet aortic valve prosthesis was implanted. Due to limited surgical exposure, valve repositioning was required, necessitating repeated cardioplegia administration and resulting in a prolonged aortic cross-clamp time (total cardiopulmonary bypass time: 365 minutes, total cross-clamp time: 262 minutes).

Attempts to wean the patient from cardiopulmonary bypass were unsuccessful. Transoesophageal echocardiography demonstrated complete left ventricular akinesia, immobile mitral and aortic valves, and minimal right ventricular contractility despite maximal inotropic support and pacing (Videos 5, 6). Conversion to full sternotomy revealed a rigid, non-compliant myocardium on palpation, consistent with SHS.

**Video 5:** Transoesophageal echocardiography video of a four-chamber view of a case with stone heart syndrome after aortic valve replacement due to severe aortic stenosis. Complete akinesia of the left heart chambers, no function of the mitral valve, and a replaced aortic valve (mechanical). The right ventricle presents a sluggish contractility after aortic valve replacement due to severe aortic stenosis on a trial to wean off cardiopulmonary bypass. No response to any pharmacological agents, and mechanical support was noted.

**Video 6:** Transoesophageal echocardiography of a three-chamber view of a case with stone heart syndrome after aortic valve replacement due to severe aortic stenosis. Complete akinesia of the left heart chambers on a trial to wean off cardiopulmonary bypass of a case with stone heart syndrome after aortic valve replacement due to severe aortic stenosis. No response to inotropic agents, mechanical support, pacing, or any devices or pharmacologic agents.

Central venous-arterial extracorporeal membrane oxygenation was initiated; however, no myocardial recovery occurred. The patient developed refractory cardiogenic shock, acute renal failure, and acute hepatic failure. Despite maximal supportive care, she died on postoperative day three due to irreversible multiorgan failure.

## Discussion

SHS is an exceedingly rare complication of cardiac surgery [1,3]. The current literature, limited to isolated case reports [12,13] and small series [5-7,14,15], does not allow assessment of its prevalence or outcomes across different socioeconomic groups. Nevertheless, given the growing recognition of social determinants of health as modifiers of cardiovascular outcomes, future studies are needed to explore their potential influence on access to care, perioperative risk, and outcomes in rare but catastrophic complications such as SHS. Scarce literature describes it as an uncommon but fatal complication, with published cases, such as two recent neonatal cases [12] and historical cohorts [3,8,11], demonstrating death in all documented instances. In one autopsy series, 4 out of 201 (2%) post-cardiac surgery patients were found to have SHS, and all died [8].

The pathophysiology of SHS reflects the consequences of prolonged global myocardial ischemia, in which profound energy depletion and calcium overload result in irreversible myocardial stiffening and loss of contractile function [1-3,5]. Cardioplegia is effective in preventing SHS by rapidly arresting and cooling the heart, thereby reducing metabolic demand; however, once complete contracture has developed, cardioplegia cannot reverse the process [3].

When myocardial ischemic protective mechanisms are insufficient, ATP stores may be rapidly exhausted, resulting in failure of actin-myosin cross-bridge detachment following contraction. Consequently, myocardial fibers become fixed in a rigid, contracted state resembling rigor mortis. Unlike post-mortem rigor, this phenomenon occurs in vivo as a result of profound ischemia. Additionally, ischemic myocardial cells lose membrane integrity, allowing calcium ions to flood into the cytoplasm and perpetuate sustained contraction. This cascade ultimately leads to the development of SHS, which is irreversible once fully established [2,3].

The marked decline in the incidence of SHS since the 1980s can be largely attributed to major advances in cardiac surgery and myocardial protection strategies [12,14,16]. Rapid induction of cardioplegia with early myocardial cooling and electrical arrest has significantly reduced myocardial metabolic demand during periods of global ischemia. Improvements in myocardial cooling techniques and more frequent administration of cardioplegia, as well as the development of more sophisticated constituents of cardioplegia solutions, have further enhanced myocardial preservation, particularly during prolonged procedures [16].

In addition, effective relief of ventricular distension through left ventricular venting and optimized suction techniques has contributed to reduced myocardial wall tension and oxygen consumption. Maintenance of adequate cardioplegia perfusion pressure, along with improved perfusion monitoring, has further optimized myocardial protection. A deeper understanding of myocardial metabolism and ischemia-reperfusion injury, combined with improved recognition and management of inadequate myocardial arrest, especially in the presence of anatomical or pathological barriers to cardioplegia delivery, such as aortic insufficiency or significant coronary artery disease, has also played a crucial role in reducing the occurrence of this catastrophic complication [16].

Before 1980, cardioplegia practices were inconsistent with respect to composition, temperature, timing, frequency, and effectiveness of delivery, which likely contributed to the higher incidence of SHS during that era [16].

In the present case, the patient unfortunately developed SHS, likely at an early stage, although this was not recognized until full sternotomy was performed, allowing complete exposure of the heart and palpation of the left ventricle. By that time, a prolonged duration had elapsed following aortic cross-clamping. In retrospect, this patient was not an ideal candidate for a minimally invasive sternotomy, as limited exposure increased technical difficulty and prolonged myocardial ischemic time.

While the current consensus suggests that moderate obesity alone is not a contraindication to mini-sternotomy, morbid obesity represents an independent risk factor that significantly increases anesthetic and surgical risk, including perioperative complications and mortality [17].

The primary aim of this report is to emphasize the critical importance of meticulous surgical planning and minimization of myocardial ischemic time [6], particularly in patients anticipated to present intraoperative technical challenges. Although SHS is now exceedingly rare, it remains a catastrophic and incurable condition that must not be overlooked. In retrospect, induction of deeper systemic hypothermia before aortic cross-clamping may have reduced the risk of SHS in our patient [3,12,14,15]. While it is impossible to determine whether this measure alone would have altered the outcome, it remains one of the few available strategies to mitigate the risk of this devastating complication. Alternatively, despite the patient’s relatively young age, transcatheter aortic valve implantation may have represented a more suitable therapeutic option given her elevated surgical risk profile.

SHS is a clinical entity that should be recognized by all cardiac surgeons. The primary objective of the surgical team must be optimal myocardial protection during periods of anoxic arrest [3]. Despite significant advances in myocardial preservation, further research is required to elucidate the underlying pathophysiological mechanisms, predisposing factors, and potential therapeutic strategies of this devastating complication of cardiac surgery.

## Conclusions

This case highlights the persistent vulnerability of the myocardium to prolonged global ischemia, even in the era of advanced cardiac surgical techniques. SHS, while exceptionally uncommon, represents an irreversible endpoint of inadequate myocardial protection and extended ischemic exposure. Careful intraoperative decision-making, including timely conversion from minimally invasive approaches when exposure is limited, is critical to preventing catastrophic myocardial injury. Heightened awareness of predisposing factors and adherence to strict myocardial protection protocols remain central to risk mitigation, as therapeutic options are extremely limited once SHS develops.
